# Cross-Protection of Dengue Virus Infection against Congenital Zika Syndrome, Northeastern Brazil

**DOI:** 10.3201/eid2508.190113

**Published:** 2019-08

**Authors:** Celia Pedroso, Carlo Fischer, Marie Feldmann, Manoel Sarno, Estela Luz, Andrés Moreira-Soto, Renata Cabral, Eduardo Martins Netto, Carlos Brites, Beate M. Kümmerer, Jan Felix Drexler

**Affiliations:** Universidade Federal da Bahia, Salvador, Brazil (C. Pedroso, M. Sarno, E. Luz, R. Cabral, E. Martins Netto, C. Brites);; Charité-Universitätsmedizin Berlin, corporate member of Freie Universität Berlin Humbolt-Universität zu Berlin and Berlin Institute of Health, Institute of Virology, Berlin, Germany (C. Fischer, A. Moreira-Soto, J.F. Drexler);; University of Bonn Medical Centre, Bonn, Germany (M. Feldmann, B.M. Kümmerer); German Centre for Infection Research (B.M. Kümmerer, J.F. Drexler);; Martsinovsky Institute of Medical Parasitology, Tropical and Vector-Borne Diseases, Sechenov University, Moscow, Russia (J.F. Drexler)

**Keywords:** Zika virus, dengue virus, cross protection, antibody-dependent enhancement, flaviviridae, Brazil, viral plaque assay, T-cell specificity, viruses, congenital Zika syndrome

## Abstract

The Zika virus outbreak in Latin America resulted in congenital malformations, called congenital Zika syndrome (CZS). For unknown reasons, CZS incidence was highest in northeastern Brazil; one potential explanation is that dengue virus (DENV)–mediated immune enhancement may promote CZS development. In contrast, our analyses of historical DENV genomic data refuted the hypothesis that unique genome signatures for northeastern Brazil explain the uneven dispersion of CZS cases. To confirm our findings, we performed serotype-specific DENV neutralization tests in a case–control framework in northeastern Brazil among 29 Zika virus–seropositive mothers of neonates with CZS and 108 Zika virus–seropositive control mothers. Neutralization titers did not differ significantly between groups. In contrast, DENV seroprevalence and median number of neutralized serotypes were significantly lower among the mothers of neonates with CZS. Supported by model analyses, our results suggest that multitypic DENV infection may protect from, rather than enhance, development of CZS.

The 2015–2016 Zika epidemic in Brazil was associated with congenital malformations summarized as congenital Zika syndrome (CZS) (*1*–*6*). As a consequence, abortion requests and pregnancy delays increased dramatically in Brazil and all of Latin America (*7*,*8*). For unknown reasons, CZS incidence was highest in northeastern Brazil (*1*,*4*,*9*,*10*). In Asia and Africa, where Zika virus circulated for much longer than it did in the Americas, Zika virus infections have not been consistently linked to CZS development; only sporadic cases have been reported (*11*). Thus, CZS development might be affected by >1 cofactor (*9*,*12*). The hypothetical list of cofactors affecting CZS development includes maternal vaccination history (*12*), exposure to larvicides (*5*), or socioeconomic factors (*1*,*13*).

Similar to the ubiquitous dengue virus (DENV), which occurs as 4 distinct serotypes, Zika virus is a flavivirus (*14*). Secondary DENV infections can be more severe than primary infections because of antibody-dependent enhancement (i.e., heterotypic subneutralizing antibodies enhancing virus entry into poorly susceptible cells) (*15*,*16*). Zika virus infection can also be enhanced by DENV antibodies in vitro (*17*,*18*) and in mice (*19*). Thus, DENV-mediated antibody-dependent enhancement may be a major cofactor of CZS development in humans (*19*–*21*). However, antibody-dependent enhancement was not observed in experimentally Zika virus–infected nonhuman primates (*22*) and during pivotal epidemiologic studies from Brazil that assessed neither individual DENV serotypes nor microcephaly cases (*5*,*9*,*23*). In addition, DENV is ubiquitous in all regions of Brazil (*20*). Therefore, to explain the accumulation of CZS cases in northeastern Brazil, a hypothetical DENV-mediated effect enhancing CZS development would require region-specific differences in past DENV exposure. To investigate the role that preexisting DENV immunity has in CZS development, we conducted serologic testing in a nested case–control framework and analyzed historical DENV genomic data from Brazil.

## Materials and Methods

### Study Population

We compared 29 mothers of children born with CZS (cases) and 108 mothers of children born without CZS (controls) from Salvador, northeastern Brazil. All mothers had evidence of past Zika virus exposure, determined by use of ELISAs and plaque-reduction neutralization tests (PRNTs) as described previously (*13*). Samples were collected consecutively at the time of delivery from May 2015 through December 2016 at the University of Bahia Climério de Oliveira maternity ward (Appendix Figure). The study was approved by the Institutional Research Ethics Board under protocol no. 1.408.49, and all women delivering during that period accepted participation in the protocol. Age distributions of cases and controls did not differ significantly (median age 26 years for cases, interquartile range 22.0–33.5; median age 29 years for controls, interquartile range 23.3–34.0; p = 0.26 by *t*-test).

### Diagnosis of CZS

CZS was diagnosed by attending gynecologists. Lead symptoms of CZS, as defined by Moore et al. (*24*), included microcephaly and other neurologic birth defects (e.g., intracranial calcifications, ventriculomegaly, dysgenesis of the corpus callosum, Dandy-Walker–like malformations, hydranencephaly, porencephaly, hydrocephalus, severe intracranial calcifications, and decreased brain tissue) (*13*). Microcephaly was identified when the measurement of the cephalic circumference was 2 SDs below that of neonates of the corresponding gestational age, according to intergrowth charts from the World Health Organization in addition to clinical and imaging data.

### PRNTs

For the serotype-specific PRNT for DENV, we used 3 μL of heat-inactivated serum (56°C, 30 min) diluted by using Dulbecco modified Eagle medium, supplemented with 1% fetal calf serum at 1:50, 1:150, 1:450, 1:1,350, 1:4,050, and 1:12,150. We split serum dilutions into 4 equal aliquots and incubated them separately in 96-well plates with 60 PFUs of DENV serotypes 1–4 (Appendix Table 1) for 60 min at 37°C. Next, we incubated the virus/serum mixtures for 90 min at 37°C in 5% CO_2_ on Vero cells grown in 24-well plates, followed by a methylcellulose/minimum essential medium overlay (2% fetal calf serum, 1.2% final methylcellulose concentration). After incubation for 4 days (DENV-1, -3, and -4) or 5 days (DENV-2), we performed formaldehyde fixation, crystal violet staining, and plaque counting. We calculated neutralizing antibody titers by using the built-in variable slope model in GraphPad Prism 6 (GraphPad Software, LLC, https://www.graphpad.com). Any titer >1:10 that reduced DENV PFU by >90% compared with control titers was considered positive. PRNT is the standard for flavivirus serology. DENV vaccine studies commonly rely on 50% plaque reduction to determine DENV serotype-specific antibody responses (*25*). To minimize the effect of potential cross-reactivity between DENV serotypes on our results, we selected a less sensitive but highly specific 90% PRNT (PRNT_90_).

### Phylogenetic Analyses

For phylogenetic analyses, we retrieved all DENV sequences available from GenBank as of June 15, 2018, that contained information on year and place of isolation. We constructed neighbor-joining trees in MEGA7 (*26*) by using a percentage distance method, a pairwise deletion option, and 1,000 bootstrap replicates. We analyzed either the junction of the envelope and the nonstructural protein 1 (NS1) encoding regions (polyprotein gene positions 2215–2454) or a fragment of 561 nt within the NS1 region (polyprotein gene positions 2650–3210). For clarity of presentation, we excluded sequences of <0.5% mutual nucleotide sequence distance. To show different genotypes, we included selected reference strains (Appendix Table 2). All DENV genome positions given within this article refer to a DENV prototype strain available in GenBank under accession no. KC294223.

### Confirmation of DENV Strains Used for PRNT

We confirmed the designation and serotype of DENV strains applied for PRNT by Sanger sequencing of the prM-C domains using strain-specific oligonucleotide primers. These primers are available upon request.

### Statistical Analyses and Visualization of PRNT Results

To plot PRNT results, we used GraphPad Prism 6. All p values result from 2-tailed tests. For power calculations, we used OpenEpi version 3 (*27*) for 2-sided 95% CIs. Regression lines were calculated by using a least squares (ordinary) fit method.

### Model Testing

To compare the effects of different factors on CZS formation, we tested mathematical logistic regression models. Each model considered 1 defined variable to predict the binary outcome as CZS case or control. We included for testing binary predictor variables such as presence or absence of DENV-1 neutralization, as well as ordinal (e.g., number of neutralized DENV serotypes) or continuous predictor variables, such as DENV-1 PRNT titers. Cases were coded as 1 and controls as 0. We fitted 15 models by using the generalized linear model function of R version 3.5.2 (https://www.r-project.org). To compare different models, we calculated the Akaike information criterion (AIC), the difference between a given and the best-supported model in AIC, and the Akaike weights by using the bbmle package version 1.0.20 in R. To show which models allow significant CZS case prediction, we calculated likelihood ratio tests for each model, and to show the effect strength of the models, we calculated odds ratios.

## Results

**Figure 1:**
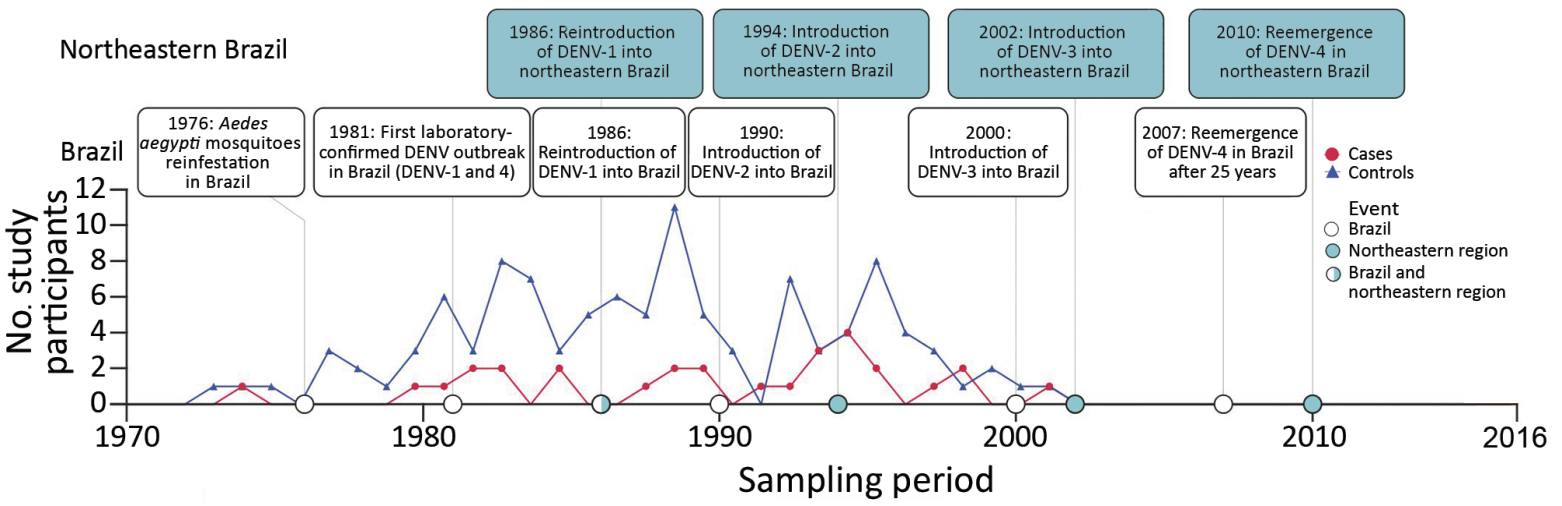
Timeline of dengue virus introduction in Brazil and birth years of participants in study of dengue virus cross-protection against congenital Zika syndrome, northeastern Brazil. DENV, dengue virus.

**Figure 2:**
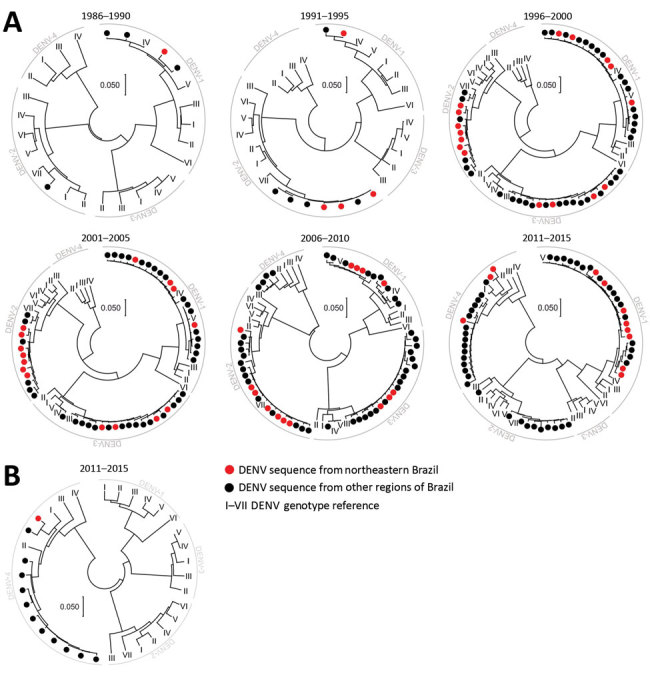
Phylogenies of dengue virus strains from Brazil. Strains circulating in northeastern and remaining Brazil are grouped in intervals of 5 years. A) Envelope-NS1 junction phylogeny. Roman numerals indicate reference sequences for relevant DENV genotypes (Appendix Table 2). DENV-2 genotypes identified by Roman numerals represent the following geographic designations: I, Asian I; II, Asian II; III, American; IV, cosmopolitan I; V, cosmopolitan II; VI, cosmopolitan III; VII, Asian/American. B) NS1 phylogeny for 2011–2015. Reference sequences were included. Scale bars indicate percent nucleotide distance. DENV, dengue virus; NS, nonstructural.

After the reinfestation of Brazil with the main DENV vector, *Aedes aegypti* mosquitoes, in 1976 (*28*), DENV-1 was introduced in 1986 (*29*), DENV-2 in 1990 (*30*), and DENV-3 in 2000 (*31*); DENV-4 reemerged in 2007 after an absence of 25 years of absence (*32*) (Figure 1). At most, 4 years after their first detection in other regions of Brazil, all 4 DENV serotypes were found in northeastern Brazil. In the databases, we identified 992 unique DENV sequences from Brazil that we used to analyze genomic DENV signatures hypothetically segregating the northeast and other regions in Brazil. Analyses of the envelope-NS1 junction, which is frequently used for genome-based serotyping (*33*), revealed high genetic identity of DENV strains from the northeast and other regions of Brazil during 30 years (Figure 2, panel A). A single DENV-4 clade apparently was found uniquely in northeastern Brazil during 2011–2015 (Figure 2, panel A). Nonetheless, these DENV-4 strains were closely related to strains from other regions when a different, larger, partial NS1 region was analyzed (Figure 2, panel B). In summary, our analyses showed no phylogenetic evidence for a unique DENV signature segregating northeastern Brazil from other regions.

**Figure 3:**
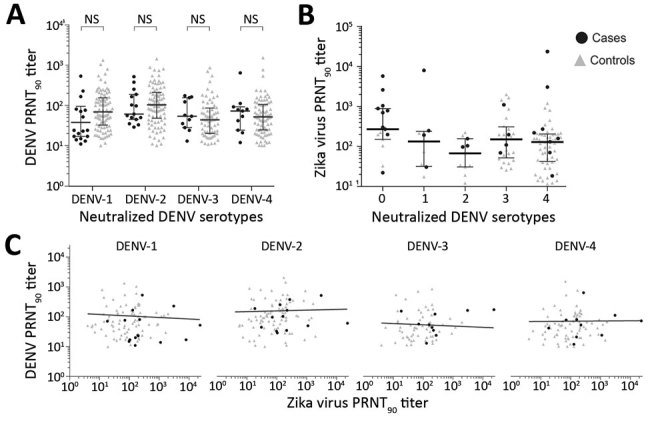
Serologic test results from participants in case–control study of cross-protection of dengue virus infection against congenital Zika syndrome, northeastern Brazil. A) Serotype-specific PRNT_90_ titers for cases and controls. Statistical significance (p<0.05) was calculated by the Mann–Whitney U test; no significance was found. B) Zika virus neutralizing antibody titers as a function of the number of neutralized DENV serotypes. C) Correlation of DENV and Zika virus titers. Statistical significance (p<0.05) was calculated by Pearson correlation; no significance was found. DENV, dengue virus; NS, not significant; PRNT_90_, 90% plaque reduction neutralization test.

Low DENV antibody titers have been shown to be a risk factor for severe disease with heterotypic DENV infection (*15*). Therefore, we analyzed the magnitude of DENV antibody titers. Overall median reciprocal PRNT_90_ titers within this study were 56.5 (95% CI 42.0–79.0) for cases and 61.4 (95% CI 54.3–73.1) for controls. Serotype-specific titers were 68.7 (95% CI 51.2–83.2) for DENV-1, 102.8 (95% CI 79.6–130.6) for DENV-2, 44.8 (95% CI 35.3–55.8) for DENV-3, and 52.6 (95% CI 41.9–66.6) for DENV-4. DENV titers did not differ significantly between cases and controls or between serotypes (Figure 3, panel A). However, we have previously shown that Zika virus antibody titers are significantly higher among mothers of neonates with CZS than among mothers of neonates without evidence of CZS (*34*), hypothetically affecting DENV antibody titer estimates. In our cohorts, Zika virus titers did not correlate with DENV titers (Figure 3, panel B) or with the number of neutralized DENV serotypes (p = 0.8459 by analysis of variance) (Figure 3, panel C), suggesting robustness of our results irrespective of individual Zika virus PRNT titers.

**Figure 4:**
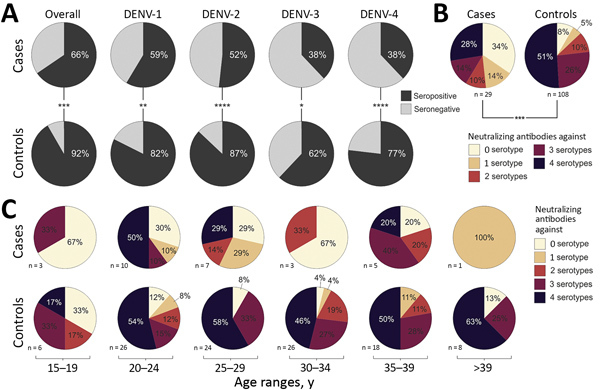
Dengue virus prevalence and neutralization among participants in case–control study of cross-protection of dengue virus infection against congenital Zika syndrome, northeastern Brazil. A) Serotype-specific DENV seroprevalence in cases and controls. Statistical significance was calculated by χ^2^ test. *p<0.05; **p<0.01; ***p<0.001; ****p<0.0001. B) Multitypic DENV neutralization in cases and controls. Statistical significance for the numbers of neutralized DENV serotypes was calculated by using the Mann–Whitney U test. ***p = 0.0004. C) Multitypic DENV neutralization in cases and controls in different age groups. DENV, dengue virus.

Strikingly, the overall DENV seroprevalence was significantly lower among cases, at 65.5%, than among controls, at 91.7% (p = 0.0003 by χ^2^ test; power 90.4%). For each DENV serotype, seroprevalence was also consistently higher among cases than controls (Figure 4, panel A). The relatively lower seroprevalence of DENV-3 and DENV-4 compared with DENV-1 and DENV-2 among study participants is consistent with the shorter circulation of these viruses in Brazil (Figure 1), again suggesting robustness of our data. Last, the median number of neutralized DENV serotypes was significantly lower among cases than among controls (p<0.0004 by Mann-Whitney U test; power 94.8%) (Figure 4, panel B). Only 27.6% of cases, compared with 50.9% of controls, had neutralizing antibodies against all 4 serotypes. Predominance of multitypic DENV exposure among controls over cases was consistently observed among participants in all age groups (Figure 4, panel C).

We conducted generalized linear model analyses to compare the effects of various factors on CZS formation. For model analysis, we considered factors that differed significantly between cases and controls in bivariate comparisons and factors that did not differ significantly. Factors included the presence and titers of neutralizing antibodies against specific DENV serotypes and the overall number of neutralized serotypes. We created 15 logistic regression models, each considering 1 factor potentially affecting CZS development (Table). With our data applied, the models considering neutralization of >2 DENV serotypes (AIC = 130.4) or the cumulative number of neutralized DENV serotypes showed the highest support (AIC = 130.6). Regarding the presence of serotype-specific neutralizing antibodies, models considering DENV-4 (AIC = 131.4) and DENV-2 (AIC = 131.5) neutralization showed the best support. Models considering age or antibody titers showed relatively lower support with AICs >140. Of all tested models, the model considering neutralization of >2 DENV serotypes showed the highest reduction of CZS risk by 84.2% (95% CI 60.5%–93.8%). Of those models considering nonbinary factors, the cumulative number of neutralized DENV serotypes showed the highest reduction of CZS risk by 42.3% (95% CI 23.7%–56.8%) per increase of neutralized serotype. The model considering neutralization of 1 DENV serotype only as a risk factor was not supported statistically or by AIC.

**Table:** Comparison of models used to identify factors affecting development of congenital Zika syndrome in study of dengue virus cross-protection against congenital Zika syndrome, northeastern Brazil*

Model	Predictor scale	AIC	ΔAIC	AW	Odds ratio (95% CI)	p value
Neutralization of >2 DENV serotypes	Binary	130.4	0	0.2812	0.158 (0.062–0.395)	<0.0001
No. neutralized serotypes	Ordinal, 5 ranks	130.6	0.2	0.2590	0.577 (0.432–0.763)	<0.0001
DENV-4 neutralization	Binary	131.4	0.9	0.1750	0.192 (0.078–0.449)	<0.0001
DENV-2 neutralization	Binary	131.5	1.1	0.1657	0.170 (0.068–0.423)	<0.0001
Neutralization of >3 DENV serotypes	Binary	133.0	2.6	0.0771	0.210 (0.086–0.492)	<0.0001
Neutralization of >1 DENV serotypes	Binary	134.8	4.4	0.0319	0.170 (0.060–0.477)	<0.0001
DENV-1 neutralization	Binary	139.1	8.6	0.0038	0.298 (0.122–0.733)	0.009
DENV-3 neutralization	Binary	140.3	9.9	0.0020	0.368 (0.154–0.844)	0.018
Neutralization of 4 DENV serotypes	Binary	140.5	10.1	0.0018	0.361 (0.139–0.852)	0.020
Anti-DENV-2 PRNT_90_ titer	Continuous	141.8	11.4	<0.001	0.998 (0.990–1.000)	0.043
Anti-DENV-1 PRNT_90_ titer	Continuous	142.8	12.4	<0.001	0.997 (0.990–1.000)	0.079
Neutralization of 1 DENV serotype	Binary	143.2	12.8	<0001	3.328 (0.776–13.477)	0.101
Age of mothers when giving birth	Continuous	143.2	12.8	<0.001	0.953 (0.896–1.010)	0.136
Anti-DENV-3 PRNT_90_ titer	Continuous	144.2	13.8	<0.001	0.996 (0.990–1.000)	0.195
Anti-DENV-4 PRNT_90_ titer	Continuous	144.9	14.5	<0.001	0.998 (0.990–1.000)	0.326

*p values were calculated by likelihood ratio tests of the different models. Models are sorted by AIC, which is an estimator of the model’s quality; models with lower AIC values are superior to models with higher AIC values. The scale of predictor variables must be considered when comparing ORs of different models. AIC, Akaike information criterion; AW, Akaike weight; DENV 1–4, dengue virus types 1–4; OR, odds ratio; PRNT_90_, 90% plaque reduction neutralization test; ΔAIC, difference between a given and the best-supported model in AIC.

## Discussion

Contrary to a large body of in vitro data, our epidemiologic data strongly suggest cross-protection from CZS development by multitypic DENV immunity. The protective effect was observed in bivariate comparisons and in model analyses. Our interpretation is consistent with anecdotal evidence reporting near-complete lack of DENV activity during the Zika virus epidemic, hypothetically resulting from cross-protection induced by previous Zika virus exposure (*35*). Moreover, in experimentally infected nonhuman primates, preexisting DENV immunity caused relatively faster clearance of Zika viremia (*22*). Strong support for our interpretation is provided by 2 recently published epidemiologic studies from Brazil and Nicaragua (*36*,*37*). In both studies, preexisting DENV immunity significantly reduced the risk for symptomatic Zika virus infection. Although those studies did not examine the effect of serotype-specific antibodies, in several epidemiologic studies, multitypic DENV immunity was cross-protective for postsecondary DENV infections (*15*,*38*,*39*). In our study, the relatively stronger cross-protection by neutralization of DENV-2 and DENV-4 may suggest that recent DENV infection boosts cross-protection against CZS because both serotypes reportedly were the predominant serotypes in northeastern Brazil before the Zika virus outbreak (*40*).

Antibody protection against DENV is related to antibody titers, and low titers are a risk factor for severe dengue (*15*). In our study, putative DENV-mediated cross-protection against CZS was apparently not linked to antibody titers. Thus, cross-protection from CZS may be mediated by immune responses (*41*) other than cross-protective antibodies. In humans, preexisting DENV immunity has been shown to boost CD4^+^ and CD8^+^ T-cell responses during Zika virus infections (*42*–*44*). In pregnant mice, DENV cross-reactive CD8^+^ T cells have been shown to be a key component of protection from fetal injury or demise during Zika virus infection (*45*,*46*). Of note, CD8^+^ T cells form a part of the placental barrier that protects the fetus from vertically acquired infections. DENV-primed CD8+ T cells might provide cross-protection from CZS at the placental barrier (*45*). As T-cell–mediated DENV cross-protection wanes over time (*39*,*45*), consecutive heterotypic DENV infections might have afforded relatively stronger and prolonged cross-protection from CZS in controls.

Our study was limited by the absence of longitudinal samples, thereby preventing definite assessments of identical DENV serostatus at the time of congenital Zika virus infection compared with the time of testing at delivery. Nonetheless, the uniformity of our results and low DENV activity during the Zika epidemic (*1*,*35*) speak against putative DENV exposure of mothers after the time of congenital Zika virus infection. Because sampling was conducted at delivery, we could not assess the time of maternal and potential congenital Zika virus infection, which affects CZS development (*2*). Because the dates of deliveries were similar among cases and controls sampled continuously within Salvador during the peak of the Zika outbreak (*1*), it seems plausible that cases and controls acquired Zika virus infection at similar stages of pregnancy (i.e., cases were probably not exclusively infected during the first trimester of pregnancy, which is most critical for CZS formation, compared with putatively later times of maternal infection in controls). Because of the small sample sizes, we could not perform PRNT for other endemic flaviviruses (e.g., yellow fever virus) that may also affect CZS development (*19*). However, northeastern Brazil has not consistently implemented yellow fever vaccination, and samples were collected before the large yellow fever outbreak that struck Brazil in the aftermath of the Zika epidemic (*47*). The comparison of historic DENV circulation in northeastern Brazil and other regions of the country is limited by incomplete genome coverage, sampling biases, and resolution of the phylogenetic trees. Nevertheless, our results match the cornerstones of DENV circulation in Brazil and the dataset is larger than other virus databases. The strengths of our study include the combination of highly specific serotype-discriminating DENV PRNT_90_ for examination of preexisting DENV immunity with serologically well-characterized samples from the most relevant persons (i.e., cases and controls sampled during the same time and in the same region) (*13*,*34*), model selection analyses, and an analysis of historical DENV exposure in Brazil.

Our data do not exclude the possibility of sporadic enhancement of CZS development by monotypic DENV immunity or subneutralizing antibodies from nonrecent exposure to DENV depending on the combination (*48*) and the chronologic sequence (*49*) of previous flavivirus infection and the time since previous flavivirus infections (*50*). However, our study strongly suggests a complex interaction between Zika virus and DENV immunity and a protective effect of strong preexisting multitypic DENV immunity of the mother on CZS development in the fetus during the Zika virus outbreak in northeastern Brazil.

Appendix Additional data for case–control study of cross-protection of dengue virus infection against congenital Zika syndrome, northeastern Brazil.

## References

[1] Netto EM, Moreira-Soto A, Pedroso C, Höser C, Funk S, Kucharski AJ, et al. High Zika virus seroprevalence in Salvador, northeastern Brazil limits the potential for further outbreaks. MBio. 2017;8:e01390–17. 10.1128/mBio.01390-17 29138300 PMC5686533

[2] Brasil P, Pereira JP Jr, Moreira ME, Ribeiro Nogueira RM, Damasceno L, Wakimoto M, et al. Zika virus infection in pregnant women in Rio de Janeiro. N Engl J Med. 2016;375:2321–34. 10.1056/NEJMoa1602412 26943629 PMC5323261

[3] França GV, Schuler-Faccini L, Oliveira WK, Henriques CM, Carmo EH, Pedi VD, et al. Congenital Zika virus syndrome in Brazil: a case series of the first 1501 livebirths with complete investigation. Lancet. 2016;388:891–7. 10.1016/S0140-6736(16)30902-3 27372398

[4] de Oliveira WK, de França GVA, Carmo EH, Duncan BB, de Souza Kuchenbecker R, Schmidt MI. Infection-related microcephaly after the 2015 and 2016 Zika virus outbreaks in Brazil: a surveillance-based analysis. Lancet. 2017;390:861–70. 10.1016/S0140-6736(17)31368-5 28647172

[5] de Araújo TVB, Ximenes RAA, Miranda-Filho DB, Souza WV, Montarroyos UR, de Melo APL, et al.; investigators from the Microcephaly Epidemic Research Group; Brazilian Ministry of Health; Pan American Health Organization; Instituto de Medicina Integral Professor Fernando Figueira; State Health Department of Pernambuco. Association between microcephaly, Zika virus infection, and other risk factors in Brazil: final report of a case-control study. Lancet Infect Dis. 2018;18:328–36. 10.1016/S1473-3099(17)30727-2 29242091 PMC7617036

[6] Cugola FR, Fernandes IR, Russo FB, Freitas BC, Dias JL, Guimarães KP, et al. The Brazilian Zika virus strain causes birth defects in experimental models. Nature. 2016;534:267–71. 10.1038/nature18296 27279226 PMC4902174

[7] Aiken AR, Scott JG, Gomperts R, Trussell J, Worrell M, Aiken CE. Requests for abortion in Latin America related to concern about Zika virus exposure. N Engl J Med. 2016;375:396–8. 10.1056/NEJMc1605389 27331661 PMC5026851

[8] Mayor S. Abortion requests increase in Latin America after Zika warning, figures show. BMJ. 2016;353:i3492. 10.1136/bmj.i3492 27334910

[9] Campos MC, Dombrowski JG, Phelan J, Marinho CRF, Hibberd M, Clark TG, et al. Zika might not be acting alone: Using an ecological study approach to investigate potential co-acting risk factors for an unusual pattern of microcephaly in Brazil. PLoS One. 2018;13:e0201452. 10.1371/journal.pone.0201452 30110370 PMC6093667

[10] Faria NR, Quick J, Claro IM, Thézé J, de Jesus JG, Giovanetti M, et al. Establishment and cryptic transmission of Zika virus in Brazil and the Americas. Nature. 2017;546:406–10. 10.1038/nature22401 28538727 PMC5722632

[11] Pettersson JH, Bohlin J, Dupont-Rouzeyrol M, Brynildsrud OB, Alfsnes K, Cao-Lormeau VM, et al. Re-visiting the evolution, dispersal and epidemiology of Zika virus in Asia. Emerg Microbes Infect. 2018;7:79. 10.1038/s41426-018-0082-5 29739925 PMC5940881

[12] Butler D. Brazil asks whether Zika acts alone to cause birth defects. Nature. 2016;535:475–6. 10.1038/nature.2016.20309 27466104

[13] Moreira-Soto A, Cabral R, Pedroso C, Eschbach-Bludau M, Rockstroh A, Vargas LA, et al. Exhaustive TORCH pathogen diagnostics corroborate Zika virus etiology of congenital malformations in northeastern Brazil. MSphere. 2018;3:e00278–18. 10.1128/mSphere.00278-18 30089647 PMC6083096

[14] Moureau G, Cook S, Lemey P, Nougairede A, Forrester NL, Khasnatinov M, et al. New insights into flavivirus evolution, taxonomy and biogeographic history, extended by analysis of canonical and alternative coding sequences. PLoS One. 2015;10:e0117849. 10.1371/journal.pone.0117849 25719412 PMC4342338

[15] Katzelnick LC, Gresh L, Halloran ME, Mercado JC, Kuan G, Gordon A, et al. Antibody-dependent enhancement of severe dengue disease in humans. Science. 2017;358:929–32. 10.1126/science.aan6836 29097492 PMC5858873

[16] Halstead SB, Mahalingam S, Marovich MA, Ubol S, Mosser DM. Intrinsic antibody-dependent enhancement of microbial infection in macrophages: disease regulation by immune complexes. Lancet Infect Dis. 2010;10:712–22. 10.1016/S1473-3099(10)70166-3 20883967 PMC3057165

[17] Li M, Zhao L, Zhang C, Wang X, Hong W, Sun J, et al. Dengue immune sera enhance Zika virus infection in human peripheral blood monocytes through Fc gamma receptors. PLoS One. 2018;13:e0200478. 10.1371/journal.pone.0200478 30044839 PMC6059439

[18] Hermanns K, Göhner C, Kopp A, Schmidt A, Merz WM, Markert UR, et al. Zika virus infection in human placental tissue explants is enhanced in the presence of dengue virus antibodies in-vitro. Emerg Microbes Infect. 2018;7:198. 10.1038/s41426-018-0199-6 30504926 PMC6274641

[19] Bardina SV, Bunduc P, Tripathi S, Duehr J, Frere JJ, Brown JA, et al. Enhancement of Zika virus pathogenesis by preexisting antiflavivirus immunity. Science. 2017;356:175–80. 10.1126/science.aal4365 28360135 PMC5714274

[20] Castanha PMS, Nascimento EJM, Braga C, Cordeiro MT, de Carvalho OV, de Mendonça LR, et al. Dengue virus-specific antibodies enhance Brazilian Zika virus infection. J Infect Dis. 2017;215:781–5. 28039355 10.1093/infdis/jiw638 PMC5854042

[21] Priyamvada L, Hudson W, Ahmed R, Wrammert J. Humoral cross-reactivity between Zika and dengue viruses: implications for protection and pathology. Emerg Microbes Infect. 2017;6:e33. 10.1038/emi.2017.42 28487557 PMC5520485

[22] Pantoja P, Pérez-Guzmán EX, Rodríguez IV, White LJ, González O, Serrano C, et al. Zika virus pathogenesis in rhesus macaques is unaffected by pre-existing immunity to dengue virus. Nat Commun. 2017;8:15674. 10.1038/ncomms15674 28643775 PMC5490051

[23] Terzian ACB, Schanoski AS, Mota MTO, da Silva RA, Estofolete CF, Colombo TE, et al. Viral load and cytokine response profile does not support antibody-dependent enhancement in dengue-primed Zika virus-infected patients. Clin Infect Dis. 2017;65:1260–5. 10.1093/cid/cix558 29017246 PMC5849103

[24] Moore CA, Staples JE, Dobyns WB, Pessoa A, Ventura CV, Fonseca EB, et al. Characterizing the pattern of anomalies in congenital Zika syndrome for pediatric clinicians. JAMA Pediatr. 2017;171:288–95. 10.1001/jamapediatrics.2016.3982 27812690 PMC5561417

[25] Kirkpatrick BD, Whitehead SS, Pierce KK, Tibery CM, Grier PL, Hynes NA, et al. The live attenuated dengue vaccine TV003 elicits complete protection against dengue in a human challenge model. Sci Transl Med. 2016;8:330ra36. 10.1126/scitranslmed.aaf1517 27089205

[26] Kumar S, Stecher G, Tamura K. MEGA7: Molecular Evolutionary Genetics Analysis version 7.0 for bigger datasets. Mol Biol Evol. 2016;33:1870–4. 10.1093/molbev/msw054 27004904 PMC8210823

[27] Sullivan KM, Dean A, Soe MM. OpenEpi: a web-based epidemiologic and statistical calculator for public health. Public Health Rep. 2009;124:471–4. 10.1177/003335490912400320 19445426 PMC2663701

[28] Pan American Health Organization. The feasibility of eradicating *Aedes aegypti* in the Americas. Rev Panam Salud Publica. 1997;1:68–72. 10.1590/S1020-49891997000100023 9128110

[29] Schatzmayr HG, Nogueira RM, Travassos da Rosa AP. An outbreak of dengue virus at Rio de Janeiro—1986. Mem Inst Oswaldo Cruz. 1986;81:245–6. 10.1590/S0074-02761986000200019 3587006

[30] Nogueira RM, Miagostovich MP, Lampe E, Souza RW, Zagne SM, Schatzmayr HG. Dengue epidemic in the stage of Rio de Janeiro, Brazil, 1990-1: co-circulation of dengue 1 and dengue 2 serotypes. Epidemiol Infect. 1993;111:163–70. 10.1017/S0950268800056788 8348928 PMC2271204

[31] Nogueira RM, Miagostovich MP, de Filippis AM, Pereira MA, Schatzmayr HG. Dengue virus type 3 in Rio de Janeiro, Brazil. Mem Inst Oswaldo Cruz. 2001;96:925–6. 10.1590/S0074-02762001000700007 11685256

[32] Figueiredo RM, Naveca FG, Bastos MS, Melo MN, Viana SS, Mourão MP, et al. Dengue virus type 4, Manaus, Brazil. Emerg Infect Dis. 2008;14:667–9. 10.3201/eid1404.071185 18394292 PMC2570911

[33] Kurolt IC, Betica-Radić L, Daković-Rode O, Franco L, Zelená H, Tenorio A, et al. Molecular characterization of dengue virus 1 from autochthonous dengue fever cases in Croatia. Clin Microbiol Infect. 2013;19:E163–5. 10.1111/1469-0691.12104 23279586

[34] Moreira-Soto A, Sarno M, Pedroso C, Netto EM, Rockstroh A, Luz E, et al. Evidence for congenital Zika virus infection from neutralizing antibody titers in maternal sera, northeastern Brazil. J Infect Dis. 2017;216:1501–4. 10.1093/infdis/jix539 29272526 PMC5853373

[35] Ribeiro GS, Kikuti M, Tauro LB, Nascimento LCJ, Cardoso CW, Campos GS, et al.; Salvador Arboviral Research Group. Does immunity after Zika virus infection cross-protect against dengue? Lancet Glob Health. 2018;6:e140–1. 10.1016/S2214-109X(17)30496-5 29389533 PMC13032803

[36] Rodriguez-Barraquer I, Costa F, Nascimento EJM, Nery N, Castanha PMS, Sacramento GA, et al. Impact of preexisting dengue immunity on Zika virus emergence in a dengue endemic region. Science. 2019;363:607–10. 10.1126/science.aav6618 30733412 PMC8221194

[37] Gordon A, Gresh L, Ojeda S, Katzelnick LC, Sanchez N, Mercado JC, et al. Prior dengue virus infection and risk of Zika: A pediatric cohort in Nicaragua. PLoS Med. 2019;16:e1002726. 10.1371/journal.pmed.1002726 30668565 PMC6342296

[38] Olkowski S, Forshey BM, Morrison AC, Rocha C, Vilcarromero S, Halsey ES, et al. Reduced risk of disease during postsecondary dengue virus infections. J Infect Dis. 2013;208:1026–33. 10.1093/infdis/jit273 23776195 PMC3749012

[39] Uno N, Ross TM. Dengue virus and the host innate immune response. Emerg Microbes Infect. 2018;7:167. 10.1038/s41426-018-0168-0 30301880 PMC6177401

[40] Salles TS, da Encarnação Sá-Guimarães T, de Alvarenga ESL, Guimarães-Ribeiro V, de Meneses MDF, de Castro-Salles PF, et al. History, epidemiology and diagnostics of dengue in the American and Brazilian contexts: a review. Parasit Vectors. 2018;11:264. 10.1186/s13071-018-2830-8 29690895 PMC5937836

[41] Collins M, de Silva A. Host response: Cross-fit T cells battle Zika virus. Nat Microbiol. 2017;2:17082. 10.1038/nmicrobiol.2017.82 28540930

[42] Grifoni A, Pham J, Sidney J, O’Rourke PH, Paul S, Peters B, et al. Prior dengue virus exposure shapes T cell immunity to Zika virus in humans. J Virol. 2017;91:e01469–17. 10.1128/JVI.01469-17 28978707 PMC5709580

[43] Delgado FG, Torres KI, Castellanos JE, Romero-Sánchez C, Simon-Lorière E, Sakuntabhai A, et al. Improved immune responses against Zika virus after sequential dengue and Zika virus infection in humans. Viruses. 2018;10:E480. 10.3390/v10090480 30205518 PMC6164826

[44] Saron WAA, Rathore APS, Ting L, Ooi EE, Low J, Abraham SN, et al. Flavivirus serocomplex cross-reactive immunity is protective by activating heterologous memory CD4 T cells. Sci Adv. 2018;4:eaar4297. **PMID: 29978039** 10.1126/sciadv.aar4297 PMC6031378 29978039

[45] Regla-Nava JA, Elong Ngono A, Viramontes KM, Huynh AT, Wang YT, Nguyen AT, et al. Cross-reactive Dengue virus-specific CD8^+^ T cells protect against Zika virus during pregnancy. Nat Commun. 2018;9:3042. 10.1038/s41467-018-05458-0 30072692 PMC6072705

[46] Wen J, Tang WW, Sheets N, Ellison J, Sette A, Kim K, et al. Identification of Zika virus epitopes reveals immunodominant and protective roles for dengue virus cross-reactive CD8^+^ T cells. Nat Microbiol. 2017;2:17036. 10.1038/nmicrobiol.2017.36 28288094 PMC5918137

[47] Moreira-Soto A, Torres MC, Lima de Mendonça MC, Mares-Guia MA, Dos Santos Rodrigues CD, Fabri AA, et al. Evidence for multiple sylvatic transmission cycles during the 2016-2017 yellow fever virus outbreak, Brazil. Clin Microbiol Infect. 2018;24:1019.e1–4. 10.1016/j.cmi.2018.01.026 29427798

[48] Guzman MG, Alvarez A, Vazquez S, Alvarez M, Rosario D, Pelaez O, et al. Epidemiological studies on dengue virus type 3 in Playa municipality, Havana, Cuba, 2001-2002. Int J Infect Dis. 2012;16:e198–203. 10.1016/j.ijid.2011.09.026 22277259

[49] Katzelnick LC, Harris E; Participants in the Summit on Dengue Immune Correlates of Protection. Immune correlates of protection for dengue: State of the art and research agenda. Vaccine. 2017;35:4659–69. 10.1016/j.vaccine.2017.07.045 28757058 PMC5924688

[50] OhAinle M, Balmaseda A, Macalalad AR, Tellez Y, Zody MC, Saborío S, et al. Dynamics of dengue disease severity determined by the interplay between viral genetics and serotype-specific immunity. Sci Transl Med. 2011;3:114ra128. 10.1126/scitranslmed.3003084 22190239 PMC4517192

