# Supplementary material for: Cross-Protection of Dengue Virus Infection against Congenital Zika Syndrome, Northeastern Brazil
Source: Emerg Infect Dis. 2019 Aug;25(8):1485–93. doi: 10.3201/eid2508.190113 (PMC6649334; doi:10.3201/eid2508.190113)
Supplement: Appendix — Additional data for case–control study of cross-protection of dengue virus infection against congenital Zika syndrome, northeastern Brazil. [file 19-0113-Techapp-s1.pdf]

Article DOI: https://doi.org/10.3201/eid2508.190113

Cross-Protection of Dengue Virus Infection against Congenital Zika Syndrome, Northeastern Brazil

Appendix

Appendix Table 1. Dengue viruses used for PRNT

Serotype	Strain	Genotype	Origin
Dengue virus 1	16007	II	Thailand
Dengue virus 2	16681	Asian I	Thailand
Dengue virus 3	H87	V	Philippines
Dengue virus 4	H241	I	Philippines

Appendix Table 2. Strains used as references for phylogenetic analyses

Serotype	Genotype	Accession number
Dengue virus 1	I	AB074760
Dengue virus 1	II	AF180818
Dengue virus 1	III	AB074761
Dengue virus 1	IV	AF226685
Dengue virus 1	V	GU131863
Dengue virus 1	VI	KR919820
Dengue virus 2	Asian I (I)	GQ868591
Dengue virus 2	Asian II (II)	JF730050
Dengue virus 2	American (III)	AF100467
Dengue virus 2	Cosmopolitan I (IV)	FJ882602
Dengue virus 2	Cosmopolitan II (V)	GQ398258
Dengue virus 2	Cosmopolitan III (VI)	JF327392
Dengue virus 2	Asian/American (VII)	KC294223
Dengue virus 3	I	AY648961
Dengue virus 3	II	EU367962
Dengue virus 3	III	FJ562107
Dengue virus 3	IV	AY146762
Dengue virus 3	V	AF317645
Dengue virus 4	I	AF289029
Dengue virus 4	II	JX024758
Dengue virus 4	III	AY618988
Dengue virus 4	IV	EF457906

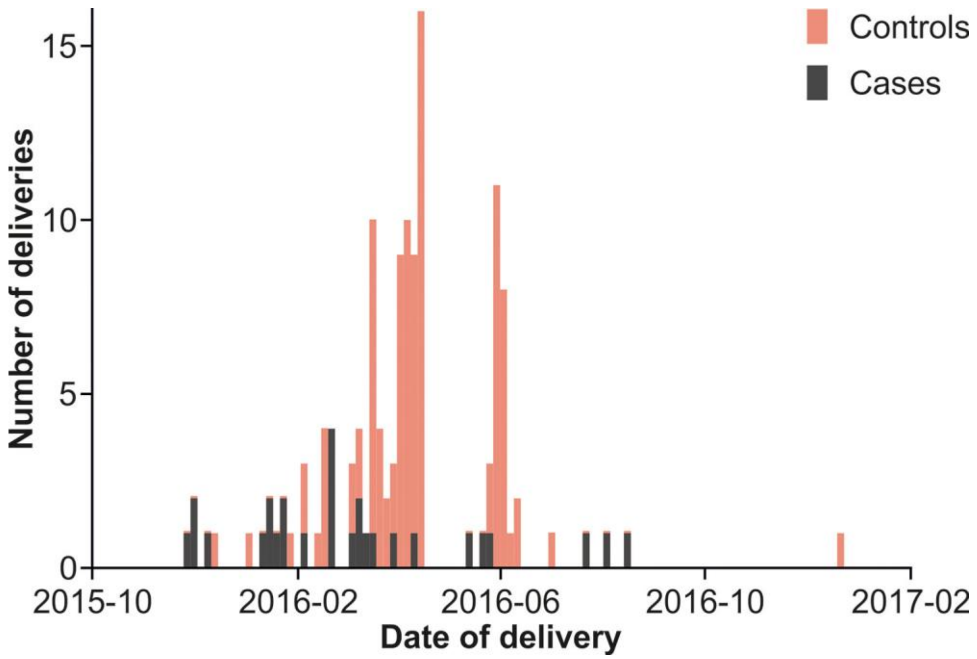

Appendix Figure. Dates of birth for controls and cases.
